# Lifestyle and Health Characteristics of the Adult Population of Serbia with Type 2 Diabetes Mellitus

**DOI:** 10.3390/medicina62040740

**Published:** 2026-04-13

**Authors:** Elijah Kiprono Toroitich, Olgica Mihaljevic, Snezana Radovanovic, Ivana Simic Vukomanovic, Jovana Radovanovic Selakovic, Viktor Selakovic, Mateja Zdravkovic, Nebojsa Zdravkovic, Vladislava Stojic, Svetlana Radevic, Katarina Janicijevic, Milos Stepovic, Melanija Tepavcevic, Simonida Delic, Dejan Jeremic

**Affiliations:** 1 Faculty of Medical Sciences, University of Kragujevac, 34000 Kragujevac, Serbia; telijahkiprono@gmail.com; 2 Health Center Novi Knezevac, 23330 Novi Knezevac, Serbia; 3 Department of Pathophysiology, Faculty of Medical Sciences, University of Kragujevac, 34000 Kragujevac, Serbia; vrndic07@yahoo.com; 4 Department of Social Medicine, Faculty of Medical Sciences, University of Kragujevac, 34000 Kragujevac, Serbia; jovanarad@yahoo.com (S.R.); drivanasimic@gmail.com (I.S.V.); cecaradevic@yahoo.com (S.R.); kaja.andreja@yahoo.com (K.J.); 5 Institute of Public Health Kragujevac, 34000 Kragujevac, Serbia; 6 Department of Epidemiology, Faculty of Medical Sciences, University of Kragujevac, 34000 Kragujevac, Serbia; 7 Department of Communication Skills, Ethics, and Psychology, Faculty of Medical Sciences, University of Kragujevac, 34000 Kragujevac, Serbia; selakovicviktor@gmail.com (V.S.); mzdravkovic39@gmail.com (M.Z.); 8 Department of Medical Statistics and Informatics, Faculty of Medical Sciences, University of Kragujevac, 34000 Kragujevac, Serbia; nzdravkovic@gmail.com (N.Z.); vladislavastojic@gmail.com (V.S.); 9 Department of Anatomy, Faculty of Medical Sciences, University of Kragujevac, 34000 Kragujevac, Serbia; stepovicmilos@yahoo.com (M.S.); melanijatepavcevic@yahoo.com (M.T.); simonidasd2025@kg.ac.rs (S.D.); dejananatom@yahoo.com (D.J.)

**Keywords:** diabetes mellitus type 2, adult population, national health survey, Republic of Serbia

## Abstract

*Background and Objectives*: Diabetes is one of the most common chronic non-communicable diseases and represents a major public health problem. At the global level, the epidemic character of diabetes mellitus can be attributed to an extended life expectancy but also to lifestyle. The aim of this study was to examine the sociodemographic, health, and lifestyle characteristics of adults with type 2 diabetes mellitus in Serbia. *Materials and Methods*: The research is part of the Serbian Population Health Survey conducted in the period from October to December 2019 by the Republic Statistical Office, in cooperation with the Institute of Public Health of Serbia “Dr Milan Jovanović Batut” and the Ministry of Health of the Republic of Serbia. The research instrument was standardized questionnaires constructed in accordance with the European Health Interview Survey (EHIS—European Health Interview Survey, wave 3) questionnaire, which were adapted to the specifics of our area. The research was conducted as a cross-sectional study on a representative sample of the adult population of Serbia. *Results*: Among 1138 adults with type 2 diabetes in Serbia (52.8% female; mean age 66.0 ± 11.9 years), overweight and obesity were highly prevalent (40.1% and 34.4%, respectively), with Obesity I predominating. Significant gender differences were observed: female more often reported obesity, multimorbidity, and depressive symptoms, whereas men were more physically active and more frequently overweight. Most participants were physically inactive, consumed breakfast and bread daily, and had low engagement in cycling and sports. Alcohol consumption was significantly higher in men, while dietary habits differed by gender for bread intake. These findings highlight substantial gender- and lifestyle-related disparities among adults with type 2 diabetes in Serbia. *Conclusions*: Targeted interventions promoting healthy lifestyle, physical activity, psychosocial support, and chronic disease management are urgently needed to address gender- and lifestyle-related disparities in adults with type 2 diabetes in Serbia.

## 1. Introduction

Diabetes mellitus is one of the most common chronic non-communicable diseases and a major public health problem worldwide, with 537 million people affected in 2021 and projections reaching 783 million by 2045, particularly in developing countries such as Serbia [1,2]. In Serbia, approximately 700,000 adults (12.2%) are living with diabetes, with a comparative prevalence of 9.1%, while around 36% of individuals with type 2 diabetes remain undiagnosed [1,3]. Type 2 diabetes accounts for about 95% of all cases, and its prevalence increases with age, with nearly half of affected individuals older than 65 years [3,4]. The disease is often diagnosed late, especially in older adults, when cardiovascular complications are already present. In Serbia, diabetes ranks among the leading causes of mortality and disease burden, with around 3000 deaths annually and a standardized mortality rate of 15.0 per 100,000 inhabitants, placing the country among those with moderately high mortality rates in Europe [3].

Individuals living with established type 2 diabetes mellitus require continuous and comprehensive disease management that extends beyond glycemic control to include lifestyle modification, management of comorbidities, and psychosocial support [4]. T2DM is commonly associated with obesity, physical inactivity, and unhealthy dietary patterns, as well as a high burden of multimorbidity, particularly cardiovascular diseases [5]. In addition, mental health conditions such as depression are frequently present and may adversely affect self-care behaviors, treatment adherence, and overall disease outcomes [6]. The distribution of these characteristics is influenced by sociodemographic factors such as age, gender, education, and socioeconomic status, which shape health behaviors, access to healthcare, and the ability to adhere to treatment recommendations. Older age is often associated with higher multimorbidity and functional limitations, while gender differences may affect lifestyle patterns, physical activity, and mental health status. Lower educational level and socioeconomic status are commonly linked to poorer health literacy, limited access to resources, and unhealthy lifestyle choices, all of which can negatively impact disease control and increase the risk of complications [7]. In this context, lifestyle factors such as diet, physical inactivity, and obesity play a crucial role not only in the development but also in the progression and management of T2DM, influencing glycemic control, cardiovascular risk, and overall health outcomes [8,9].

Although prevention remains important, large proportions of adults already live with T2DM and require continuous medical care and support for lifestyle management [9]. Understanding the sociodemographic, clinical, and lifestyle characteristics of individuals with established T2DM is therefore crucial for optimizing disease management, improving adherence to therapy, and reducing complications. In addition, gender-related differences in behavioral patterns, multimorbidity burden, and mental health status may significantly influence health outcomes [10]. However, comprehensive population-based data describing these characteristics in Serbia remain limited, highlighting the need for further investigation.

The aim of this study was to characterize the sociodemographic, health, and lifestyle profile of adults living with type 2 diabetes mellitus in Serbia, with particular emphasis on gender differences relevant to disease management and health outcomes.

## 2. Materials and Methods

This study is part of the Health Survey of the Population of Serbia, conducted from October to December 2019 by the Statistical Office of the Republic of Serbia, in collaboration with the Institute of Public Health of Serbia “Dr Milan Jovanović Batut” and the Ministry of Health. The survey was a cross-sectional study targeting individuals aged 15 years and older living in private households. Residents of collective or institutional settings and those unable to participate due to illiteracy, cognitive, or physical limitations were excluded.

A nationally representative, stratified, two-stage random sampling design was applied, with stratification by settlement type (urban/rural) and region (Belgrade, Vojvodina, Šumadija and Western Serbia, Southern and Eastern Serbia). Enumeration areas from the 2011 Population Census served as primary sampling units and were systematically selected with probability proportional to size; within these, households were randomly sampled to ensure geographic representativeness.

The sample size was calculated based on EUROSTAT precision requirements for the indicator “proportion of persons limited in performing daily activities” [11]. Assuming an 8% prevalence, with a desired error below 1% and 95% confidence, approximately 6000 households (~15,000 individuals) were needed to ensure nationally and regionally representative estimates. Ten households per enumeration area across 600 areas were selected, with reserves for non-response. The realized sample included 5114 households (15,621 individuals), with 12,426 participants aged 20 and above analyzed, stratified by gender and age.

Data collection adhered to EHIS—Wave 3 methodology over three months (October–December 2019), in line with recommendations that fieldwork cover at least three months including autumn. Data collection adhered to the European Health Interview Survey (EHIS)—Wave 3 methodology, according to the official methodological guidelines issued by Eurostat [11]. Ethical standards followed the Declaration of Helsinki and national legislation based on the Decision on the Official Statistics Program for the Period 2016–2020 (“Official Gazette of RS”, No. 55/2015) and the Regulation on the 2019 Official Statistics Plan (“Official Gazette of RS”, No. 105/2018), including GDPR and the Law on Official Statistics [12]. Participants received information sheets, and written informed consent was obtained. Personally identifiable data were coded, stored securely, and the results were reported in an aggregated form. The dataset was provided to the University of Kragujevac for research purposes.

Type 2 diabetes mellitus was defined based on self-reported physician diagnosis, as assessed through the EHIS questionnaire item asking participants whether they had ever been diagnosed with diabetes by a medical doctor, excluding gestational diabetes.

Body mass index (BMI) was calculated from directly measured height and weight (kg/m^2^) and categorized according to World Health Organization criteria into underweight (<18.5 kg/m^2^), normal weight (18.5–24.9 kg/m^2^), overweight (25.0–29.9 kg/m^2^), and obesity (≥30.0 kg/m^2^). Obesity was further classified into class I (30.0–34.9), class II (35.0–39.9), and class III (≥40.0). For descriptive purposes, obesity was presented both as a single category and further stratified into classes I–III.

Physical activity was assessed using the EHIS Physical Activity Questionnaire (EHIS-PAQ), which includes work-related, transport-related, and leisure-time activity domains. Activities of daily living (ADL) were measured using the ICF four-point scale (0–15), and instrumental ADL (IADL) via seven tasks (0–21), with higher scores indicating greater limitation. For analytical purposes, duration of moderate and vigorous activities was categorized according to WHO recommendations (<150 vs. ≥150 min per week).

Alcohol consumption was assessed based on self-reported frequency of intake during the past 12 months and categorized into six groups (never, not in the past 12 months, occasional, moderate, frequent, no response) for statistical analysis in order to avoid sparse cells.

Multimorbidity was defined as the presence of two or more self-reported chronic conditions diagnosed by a physician.

Depressive symptoms were assessed using the standardized EHIS depression module. Scores were calculated according to the official EHIS scoring guidelines, with higher values indicating greater symptom burden.

Socioeconomic and demographic variables included in the analysis were defined as follows: age was categorized into seven groups: 20–29, 30–39, 40–49, 50–59, 60–69, 70–79, and ≥80 years; region included Belgrade, Vojvodina, Šumadija and Western Serbia, and Southern and Eastern Serbia; marital status was classified as never married, divorced/separated/widowed, and married/cohabiting; household size was recorded as the number of household members and presented as mean ± standard deviation. Education was categorized into elementary school or less, middle school, and higher levels of education. Employment status was defined as unemployed, inactive, employed, and other. The welfare index was classified into poor, middle layer, and wealthy.

The study population consisted exclusively of respondents with self-reported type 2 diabetes mellitus. The analysis aimed to describe their sociodemographic, health, and lifestyle characteristics. Gender was used as the primary grouping variable in comparative analyses.

The Serbian version of the EHIS questionnaire was translated and culturally adapted according to Eurostat recommendations and implemented by the Institute of Public Health of Serbia in collaboration with the Statistical Office of the Republic of Serbia [13]: household info panels, individual questionnaires (≥5 years, adult/child versions), self-administered questionnaires for sensitive topics (≥15 years), and measurement forms for height, weight, and blood pressure. Collection methods included face-to-face interviews, self-administered questionnaires, and direct anthropometric measurements, using CAPI and PAPI. Instruments captured household, demographic, socioeconomic, health status, healthcare utilization, and health determinant information, including nutrition, physical activity, risk factors, and social support.

Statistical analysis included descriptive statistics (frequencies, percentages, means, and standard deviations) to summarize the sociodemographic, clinical, and lifestyle characteristics of adults with type 2 diabetes. Group differences between categorical variables were assessed using Chi-square tests for testing associations between nominal variables. Continuous variables were compared using Student’s *t*-tests, as all variables met the assumptions of normality. Significance was set at *p* < 0.05. Analyses were performed using SPSS v21.0. Since the data originate from a nationally representative, stratified, two-stage survey design, it should be noted that the analyses did not formally account for the complex survey design (e.g., sampling weights, clustering, or stratification). This limitation may affect variance estimates and statistical inference, potentially leading to underestimation or overestimation of standard errors.

## 3. Results

The study included 1138 respondents with type 2 diabetes mellitus, of whom 601 (52.8%) were female and 537 (47.2%) male. The mean age was 66.04 ± 11.85 years (range 20–99), 66.85 ± 12.57 for females and 65.15 ± 10.93 for males. Among the 12,426 participants aged ≥ 20 years in the survey, the prevalence of type 2 diabetes was 9.15%, with a higher proportion of females than males.

Overweight and obesity were highly prevalent, affecting nearly three-quarters of participants. Overweight was more common among men, while obesity was more frequent among females (*p* = 0.009) (Table 1).

Socioeconomic and demographic characteristics of the study population are summarized in Table 2. Most participants were older adults, predominantly aged 60–69 years, married or partnered, and had secondary education. The majority were inactive and belonged to lower welfare categories. Significant gender differences were observed in age distribution (*p* < 0.001), marital status (*p* < 0.001), education level (*p* < 0.001), employment status (*p* < 0.001), and welfare index (*p* = 0.012). Males were more often married and employed, whereas females were more frequently widowed or divorced and had lower educational attainment.

Statistically significant gender differences were found for the following variables: alcohol consumption (*p* < 0.001), bread consumption (*p* = 0.005), physical activity (*p* < 0.001), cycling (*p* < 0.001), and sports participation (*p* = 0.007).

Most respondents were non-smokers, and alcohol consumption was generally low to moderate, with clear gender differences. Alcohol consumption differed significantly between males and females, with men reporting higher frequencies across all categories (*p* < 0.001) (Table 3).

Most participants reported regular breakfast and daily bread consumption, while intake of milk, dairy products, and fruit was generally moderate, and overall physical activity levels were low, with the majority engaging predominantly in sedentary behavior and insufficient durations of walking, cycling, and sports activities.

Nearly 90% of males ate bread daily, while a notable proportion of females ate bread occasionally (13.3%) or never (2.3%), showing significant gender differences (*p* = 0.005). Most females performed sedentary work (48.8%), whereas men were more engaged in walking activities (45.1%) and mostly heavy physical work (7.1% vs. 2.2% in females) (*p* < 0.001). There were statistically significant gender differences in the proportion of respondents engaging in cycling (*p* < 0.001) and sports activities for more than 150 min (*p* = 0.007): males were significantly more active than females (cycling: 21% vs. 11.3%; sports: 7.4% vs. 2.8%) (Table 4).

Use of prescribed medication was very high, with no significant difference between females and males. In contrast, unprescribed medication was more commonly reported by females (*p* = 0.001). Multimorbidity was highly prevalent, particularly among females, whereas males were more likely to report a single or no chronic condition (*p* = 0.009). Most participants reported no depressive symptoms, but females more frequently experienced mild or moderate symptoms, resulting in higher average depression scores compared to males (*p* < 0.001). Among participants with obesity, first-grade obesity was most common, followed by second- and third-grade obesity, with no significant differences between genders (Table 5).

## 4. Discussion

Diabetes mellitus is a chronic disease with increasing global prevalence driven by demographic and lifestyle factors, and in Serbia, it remains a major public health burden affecting a large number of adults and contributing significantly to mortality [13,14,15,16]. This study provides an overview of adults with type 2 diabetes in Serbia, highlighting important gender and lifestyle differences: men were more often overweight and physically active, while females had higher rates of obesity, multimorbidity, and depressive symptoms. Differences were also observed in age, marital status, household size, education, employment, and welfare index, emphasizing the need for gender-sensitive approaches in disease management.

Population-based preventive measures, as outlined in Serbia’s National Program for the Prevention and Early Detection of Type 2 Diabetes, are essential [17]. Early-onset type 2 diabetes is linked to behavioral (poor diet, low physical activity, smoking), environmental (pollution, extreme temperatures), and metabolic (high BMI) risk factors, with high BMI being the leading contributor [18,19]. Our findings align with these patterns: overweight and obesity were highly prevalent, men were more often overweight, and females more frequently obese, highlighting the importance of targeting lifestyle interventions and weight management, especially in high-risk groups.

Previous research suggests that risk factor profiles differ by gender: females are more affected by high BMI, passive smoking, and low physical activity, whereas men are more influenced by active smoking and pollution [20,21,22,23]. Our study reflects these patterns, showing higher obesity and multimorbidity among females and lower physical activity compared to males, underscoring the need for gender-sensitive lifestyle interventions and targeted preventive strategies in Serbia.

Research emphasizes the need to focus on high-risk subgroups, such as younger patients and those with a lower BMI, for targeted screening and prevention [24]. In our study, physical inactivity was widespread, especially among females, and overweight and obesity were highly prevalent. These findings highlight the importance of early detection, weight management, and tailored lifestyle interventions, alongside medical treatment, to reduce diabetes-related complications in Serbia [25].

Depression and multimorbidity are known to complicate diabetes management, particularly among females [26]. In our study, females with type 2 diabetes more frequently reported depressive symptoms and multimorbidity, whereas men showed higher physical activity levels and a lower prevalence of depression. Significant gender differences were also observed in age, marital status, household size, education, employment, and welfare index. These results underscore the need for gender-sensitive interventions that integrate psychological support, chronic disease management, and promotion of healthy lifestyles, tailored to the sociodemographic context of adults with type 2 diabetes in Serbia. Special attention should be given to blood pressure control, particularly in individuals with obesity, type 2 diabetes, and higher cardiovascular risk at younger ages, alongside intensive management of other risk factors to reduce vascular comorbidities [27]. Aggressive multifactorial treatment according to current guidelines is essential for all adults with type 2 diabetes, while depression, especially among females, can undermine disease management and self-care [28]. Enhanced awareness, targeted screening, psychological support, and education for healthcare professionals and the public are crucial to address gender differences and improve outcomes [29,30].

This study has several limitations. The analyses did not account for the complex survey design, including stratification, clustering, and sampling weights, which may have affected variance estimates and statistical inference. The study had a cross-sectional design, which prevents causal inferences regarding the observed associations between sociodemographic, health, and lifestyle factors. Then, data were self-reported, which may introduce recall or reporting bias, particularly for sensitive information such as alcohol consumption and physical activity. Also, we did not perform regression analyses to adjust for potential confounders; the main focus of this study was to provide a descriptive overview of the population characteristics and gender differences, serving as a foundation for future analytical studies. Finally, the study population was limited to respondents with type 2 diabetes who participated in the survey, which may limit generalizability to all adults with diabetes in Serbia.

## 5. Conclusions

The results indicated that type 2 diabetes was most prevalent among individuals aged 60–69 years and was more common in females. Most participants were overweight or obese, with males more frequently overweight and females more often obese. Multimorbidity and depressive symptoms were more common in females, whereas males more frequently reported the absence of depressive symptoms. Physical inactivity was prevalent overall, but males were significantly more active than females. Significant gender differences were also observed in age, marital status, household size, education, employment, and welfare index. These findings highlight the need for targeted diabetes prevention and management programs, promotion of physical activity and healthy lifestyles, particularly for females, psychosocial support and monitoring of depressive symptoms, and educational and economic interventions to reduce obesity and improve material well-being. Implementing such strategies could improve health outcomes and reduce gender and socioeconomic disparities among adults with type 2 diabetes in Serbia.

## Figures and Tables

**Table 1:** Distribution of BMI categories by gender among adults with type 2 diabetes mellitus in Serbia.

BMI Category (kg/m^2^)	Total n (%)	Female n (%)	Male n (%)	*p*-Value
Underweight	3 (0.3)	2 (0.3)	1 (0.2)	χ^2^ = 11.525 *p* = 0.009 *
Normal weight	287 (25.2)	171 (28.4)	116 (21.6)	
Overweight	456 (40.1)	203 (33.8)	253 (47.1)	
Obesity	392 (34.4)	224 (37.3)	168 (31.3)	

* *p*-value calculated using the Chi-square test.

**Table 2:** Socioeconomic and demographic characteristics of the study population.

Variables		Study Population			χ^2^
		Total n = 1138	Female n = 601	Male n = 537	
		N (%)			
Age	20–29	12 (1.1%)	10 (1.7%)	2 (0.4%)	χ^2^ = 24.400 *p* < 0.001 *
	30–39	18 (1.6%)	12 (2%)	6 (1.1%)	
	40–49	72 (6.3%)	31 (5.2%)	41 (7.6%)	
	50–59	161 (14.1%)	81 (13.5%)	80 (14.9%)	
	60–69	428 (37.6%)	202 (33.6%)	226 (42.1%)	
	70–79	313 (27.5%)	178 (29.6%)	135 (25.1%)	
	80+	134 (11.8%)	87 (14.4%)	47 (8.8%)	
Region	Belgrade	261 (22.9%)	133 (22.1%)	128 (23.8%)	χ^2^ = 0.650 *p* = 0.885
	Vojvodina	283 (24.9%)	153 (25.5%)	130 (24.2%)	
	Šumadija and Western Serbia	311 (27.3%)	163 (27.1%)	148 (27.6%)	
	Southern and Eastern Serbia	283 (24.9%)	152 (25.3%)	131 (24.4%)	
Marital status	Never married/in cohabiting relationship	44 (3.9%)	17 (2.8%)	27 (5.1%)	χ^2^ = 99.117 *p* < 0.001 *
	Divorce/Separation/widowed	323 (28.4%)	246 (40.9%)	77 (14.3%)	
	Married/partnered	771 (67.8%)	338 (56.3%)	433 (80.6%)	
Household size (mean ± SD)		3.42 ± 2.06	3.29 ± 2.04	3.56 ± 2.07	*p* = 0.026 *
Education	Elementary school or less	452 (39.7%)	312 (51.9%)	140 (26.1%)	χ^2^ = 79.556 *p* < 0.001 *
	Middle school	531 (46.7%)	219 (36.4%)	312 (58.1%)	
	Higher level of education	155 (13.6%)	70 (11.7%)	85 (15.8%)	
Employment status	Unemployed	118 (10.4%)	61 (10.1%)	57 (10.6%)	χ^2^ = 24.661 *p* < 0.001 *
	Inactive	849 (74.6%)	479 (79.7%)	370 (68.9%)	
	Employed	163 (14.3%)	58 (9.7%)	105 (19.6%)	
	Other	8 (0.7%)	3 (0.5%)	5 (0.9%)	
Wealth index	Poor	500 (43.9%)	284 (47.2%)	216 (40.2%)	χ^2^ = 8.797 *p* = 0.012 *
	Middle layer	238 (20.9%)	129 (21.5%)	109 (20.3%)	
	Wealthy	400 (35.1%)	188 (31.3%)	212 (39.5%)	

* Independent samples T test for depression score and Chi-square test for other variables; values less than 0.05 indicated statistical significance.

**Table 3:** Characteristics of the study population in relation to health behavior.

Variable	Category	Total (n = 1138) N (%)	Female (n = 601) N (%)	Male (n = 537) N (%)	χ^2^/*p*
Smoking	No response	292 (25.7%)	161 (26.8%)	131 (24.4%)	χ^2^ = 3.504 *p* = 0.320
	Yes, daily	183 (16.1%)	88 (14.7%)	95 (17.7%)	
	Yes, occasionally	57 (5.0%)	32 (5.3%)	25 (4.7%)	
	No	606 (53.2%)	320 (53.2%)	286 (53.2%)	
Alcohol consumption	Never	292 (25.7%)	237 (39.4%)	55 (10.2%)	χ^2^ = 112.73 *p* < 0.001 *
	Not in the past 12 months	130 (11.4%)	48 (7.9%)	82 (15.3%)	
	Occasional (≤1 time/month)	140 (12.3%)	62 (10.3%)	78 (14.5%)	
	Moderate (2–4 times/month)	66 (5.8%)	16 (2.7%)	50 (9.3%)	
	Frequent (≥1 time/week)	130 (11.4%)	28 (4.7%)	102 (19.0%)	
Use of psychoactive substances	No response	537 (47.2%)	291 (48.4%)	246 (45.8%)	χ^2^ = 2.347 *p* = 0.504
	Yes, previously but not in the past 12 months	2 (0.2%)	0	2 (0.4%)	
	No, never	536 (47.1%)	274 (45.6%)	262 (48.8%)	

* Chi-square test for other variables; a value less than 0.05 indicated statistical significance.

**Table 4:** Dietary habits and physical activity of the study population.

Variable	Category	Total (n = 1138) N (%)	Female (n = 601) N (%)	Male (n = 537) N (%)	χ^2^/*p*
Breakfast consumption	Never	20 (1.8%)	12 (2.0%)	8 (1.5%)	χ^2^ = 0.873 *p* = 0.646
	Sometimes	113 (9.9%)	62 (10.3%)	51 (9.5%)	
	Every day	1005 (88.3%)	527 (87.7%)	478 (89.0%)	
Bread consumption	Never	19 (1.7%)	14 (2.3%)	5 (0.9%)	χ^2^ = 10.580 *p* = 0.005 *
	Sometimes	132 (11.6%)	80 (13.3%)	52 (9.7%)	
	Every day	987 (86.7%)	507 (84.4%)	480 (89.4%)	
Milk and dairy consumption	Once or more per day	452 (39.7%)	236 (39.3%)	216 (40.2%)	χ^2^ = 4.199 *p* = 0.521
	4–6 times per week	284 (25.0%)	140 (23.3%)	144 (26.8%)	
	1–3 times per week	244 (21.4%)	139 (23.1%)	105 (19.6%)	
	Less than once per week	81 (7.1%)	47 (7.8%)	34 (6.3%)	
	Never	26 (2.3%)	13 (2.2%)	13 (2.4%)	
Fruit consumption	Once or more per day	493 (43.3%)	254 (42.3%)	239 (44.5%)	χ^2^ = 0.898 *p* = 0.970
	4–6 times per week	259 (22.8%)	140 (23.3%)	119 (22.2%)	
	1–3 times per week	260 (22.8%)	140 (23.3%)	120 (22.3%)	
	Less than once per week	63 (5.5%)	35 (5.8%)	28 (5.2%)	
	Never	12 (1.1%)	6 (1.0%)	6 (1.1%)	
General physical activity	Mostly sitting or standing	492 (43.2%)	293 (48.8%)	199 (37.1%)	χ^2^ = 27.837 *p* < 0.001 *
	Mostly walking	479 (42.1%)	237 (39.4%)	242 (45.1%)	
	Mostly heavy physical work	51 (4.5%)	13 (2.2%)	38 (7.1%)	
	Not working	64 (5.6%)	31 (5.1%)	33 (6.0%)	
Walking	<150 min	973 (85.5%)	519 (86.4%)	454 (84.5%)	χ^2^ = 2.467 *p* = 0.116
	≥150 min	165 (14.5%)	82 (13.6%)	83 (15.5%)	
Cycling	<150 min	957 (84.1%)	533 (88.7%)	424 (79.0%)	χ^2^ = 21.123 *p* < 0.001 *
	≥150 min	181 (15.9%)	68 (11.3%)	113 (21.0%)	
Sports participation	<150 min	1081 (95.0%)	584 (97.2%)	497 (92.6%)	χ^2^ = 7.179 *p* = 0.007 *
	≥150 min	57 (5.0%)	17 (2.8%)	40 (7.4%)	

* Chi-square test for other variables; values less than 0.05 indicated statistical significance.

**Table 5:** Medication use, multimorbidity, depression symptoms, and BMI of the study population.

Variable	Category	Total (n = 1138) N (%)	Female (n = 601) N (%)	Male (n = 537) N (%)	χ^2^/*p*
Use of prescribed medication	Yes	1092 (96.0%)	582 (96.8%)	510 (95.0%)	χ^2^ = 1.041 *p* = 0.308
	No	46 (4.0%)	19 (3.2%)	27 (5.0%)	
Use of unprescribed medication	Yes	513 (45.1%)	299 (49.8%)	214 (39.8%)	χ^2^ = 11.160 *p* = 0.001 *
	No	625 (54.9%)	302 (50.2%)	323 (60.2%)	
Chronic illnesses	Multimorbidity	997 (87.6%)	541 (90.0%)	456 (84.9%)	χ^2^ = 6.797 *p* = 0.009 *
	One illness	141 (12.4%)	60 (10.0%)	81 (15.1%)	
Depression symptoms	No symptoms	942 (82.8%)	467 (77.7%)	475 (88.4%)	χ^2^ = 23.051 *p* < 0.001 *
	Weak symptoms	143 (12.6%)	97 (16.1%)	46 (8.6%)	
	Depressive episode	53 (4.7%)	37 (6.2%)	16 (3.0%)	
Depression score (X ± SD)		2.24 ± 3.63	2.71 ± 3.91	1.72 ± 3.21	*p* < 0.001 *
Obesity grade	First grade	256 (65.3%)	140 (62.5%)	116 (69.1%)	χ^2^ = 1.858 *p* = 0.395
	Second grade	98 (25.0%)	60 (26.8%)	38 (22.6%)	
	Third grade	38 (9.7%)	24 (10.7%)	14 (8.3%)	

* Independent samples T test for depression score and Chi-square test for other variables; value less than 0.05 indicated statistical significance.

## Data Availability

The original contributions presented in this study are included in the article. Further inquiries can be directed to the corresponding author.
